# 功能性老年乳粉中300种非法添加药物及其类似物的液相色谱-高分辨质谱分析

**DOI:** 10.3724/SP.J.1123.2023.05013

**Published:** 2023-11-08

**Authors:** Xiao NING, Shaoming JIN, Zhiyuan LI, Chongjun YANG, Da MAO, Jin CAO

**Affiliations:** 1.中国食品药品检定研究院, 北京 100050; 1. National Institutes for Food and Drug Control, Beijing 100050, China; 2.SCIEX中国, 北京 100015; 2. SCIEX China, Beijing 100015, China; 3.济宁市公安局刑事科学技术研究所, 山东 济宁 272000; 3. Criminal Science & Technology Institute of Jining Public Security Bureau, Jining 272000, China; 4.中国计量科学研究院化学计量与分析科学研究所, 北京 100029; 4. Division of Chemical Metrology and Analytical Science, National Institute of Metrology, Beijing 100029, China

**Keywords:** 超高效液相色谱, 高分辨质谱, 四极杆飞行时间质谱, 非法添加, QuEChERS, 功能性乳粉, 结构类似物, ultra-high performance liquid chromatography (UHPLC), high resolution mass spectrometry (HRMS), quadrupole-time of flight-mass spectrometry (QTOF-MS), illegally added, QuEChERS, functional milk powder, structural analogues

## Abstract

随着老龄化进程不断加剧,各类声称保健功能的老年乳粉不断涌现,一些生产企业通过非法添加化学药物来实现产品声称的显著功效,危害消费者健康。现有标准方法需要按照功能声称分类别进行检测,不仅耗费人力物力,还严重制约了日常监管工作对未知风险物质的侦查能力。本工作应用QuEChERS净化结合具有Zeno SWATH^®^全息式数据非依赖型采集定量(data-independent acquisition, DIA)技术的超高效液相色谱-四极杆飞行时间质谱,建立了适用于功能性老年乳粉中300种非法添加化学药物的高通量筛查定量分析方法及未知结构类似物识别策略。针对乳粉基质特点开发了QuEChERS净化流程,通过Kinetex F5色谱柱(100 mm×3.0 mm, 2.6 μm)分离,以5 mmol/L甲酸铵水溶液(含0.1%(v/v)甲酸)和甲醇-乙腈(1∶1, v/v)作为流动相进行梯度洗脱。从线性范围、灵敏度、基质效应、精密度和准确度等方面对定量分析方法进行验证。在建立300种已知药物定性筛查数据库的基础上,应用电子激活解离(electron-activated dissociation, EAD)碎裂技术,获得丰富的二级质谱裂解碎片信息,通过归属分析对未知风险物质进行鉴别和确证。所有已知目标药物在相应浓度范围内呈良好的线性关系,相关系数>0.99, 检出限(LOD)和LOQ分别在0.04~2.7和0.2~8.0 μg/kg范围内。3个水平下的平均加标回收率为73.1%~125.2%,相对标准偏差(RSD)≤14.8%。将该方法应用于60份实际样品中非法添加物质的检测,在两份样品中分别检出苯乙双胍及西地那非药物,并成功识别出一种安非他明结构类似物乙非他明。该方法简便、灵敏、准确,对于功能性乳粉的日常监督执法工作具有实际应用价值。

## Abstract (en)

Various types of milk powder purportedly providing diverse health functions have emerged with the growth of the country’s elderly population. Some manufacturers illegally add chemical drugs to their products to achieve their reported benefits, which poses a threat to consumer health. The existing standard methods are inapplicable to such complex sample matrices and require testing based on functional claims and classification. These limitations not only consume manpower and resources but also seriously impede daily regulatory efforts to detect unknown risk substances. In this study, a high-throughput method for the screening and quantitative analysis of 300 illegally added chemical drugs in functional milk powder and an identification strategy for unknown structural analogues were established using Zeno SWATH^®^ data-independent acquisition (DIA) ultra-high performance liquid chromatography-high resolution mass spectrometry (UHPLC-HRMS) technology combined with a QuEChERS sample purification method. The QuEChERS purification process was developed according to the characteristics of milk powder matrix. The supernatant was separated on a Kinetex F5 column (100 mm×3.0 mm, 2.6 μm) by gradient elution using 5 mmol/L ammonium formate aqueous solution (0.1% (v/v) formic acid, ) and methanol-acetonitrile (1∶1, v/v) as mobile phases. The method was validated in terms of selectivity, linearity, limits of detection and quantification (LODs and LOQs, respectively), matrix effect, accuracy, and precision. Based on a screening database for the 300 target substances, electron-activated dissociation (EAD) fragmentation was applied to obtain rich secondary MS fragmentation information, and unknown structural analogues were identified and confirmed through fragment attribution analysis. The results indicated that all compounds had good linear relationships in certain ranges with correlation coefficients >0.99. The LODs and LOQs were 0.04-2.7 and 0.2-8.0 μg/kg, respectively. The average recoveries at three spiked levels were in the range of 73.1%-125.2%, and the relative standard deviations were ≤14.8% (*n*=6). When the developed method was applied to detect illegally added chemicals in 60 functional milk powder samples, it detected benzoguanidine and sildenafil and successfully identified ethylphenidate, which is the structural analogue of an amphetamine. The proposed method is simple, sensitive, and accurate; thus, it may have practical application value for the daily supervision and law enforcement of milk powders with reported health functions.

随着我国老龄化进程不断加剧,保养保健观念逐步普及,人们对功能性食品的需求也从保健食品延伸到了普通食品^[1]^。为了迎合老年群体个性化的需求,宣称具有调节血糖、增强免疫力和改善睡眠等功能的老年乳粉不断涌现^[2]^,一些生产企业通过非法添加化学药物来实现产品声称的显著功效,对老年人的健康构成潜在威胁^[3]^。

非法添加的化学药物种类繁多,添加剂量及种类不明确^[4⇓-6]^。现有国家标准方法针对已知化学物质进行靶向分析,需要按照功能声称分类别进行检测,涉及方法种类繁多,且前处理及检测技术不尽相同,既耗费人力物力,又严重制约了监管部门对未知风险物质的侦查能力。因此,迫切需要开发一种简便、灵敏的不受类别限制的高通量分析方法,用于新兴功能性老年乳粉中非法添加化学药物的高效侦查。

高分辨质谱可以提供更高的选择性和质量精度^[7,8]^,结合色谱分离手段,可实现对异构体或具有相似精确质量数分子的准确区分^[9]^。但现有研究多针对单一或几个类别的已知药物建立筛查数据库,同时实现对数据库中已知化合物定量分析和对未知结构类似物预测确证的策略报道较少。顺序窗口采集所有理论质谱模式(sequential window acquisition of all theoretical mass spectra, SWATH)结合数据非依赖型采集(data-independent acquisition, DIA)技术克服了原有依赖型采集(data-dependent acquisition, DDA)模式的随机性和局限性,具有快速的采集速度、高质量的MS/MS数据和显著提高二级碎片灵敏度等优势^[10]^。此外,对于未知物的鉴定,区别于传统的碰撞活化解离(collision-activated dissociation, CAD)碎裂技术,新型的电子激活解离(electron-activated dissociation, EAD)碎裂技术通过独立捕获前体离子和自由电子,实现有效的自由基断裂,从而获得更丰富的碎片信息,与CAD形成良好的互补性,有效提升定性和未知物解析的准确度。上述技术赋予UHPLC-HRMS兼具小分子定性和定量分析的潜力,已逐步应用于蛋白质组学领域,但在食品领域中的应用尚待开发。

在样品前处理方面,已有研究使用有机溶剂稀释提取后直接分析^[11⇓-13]^,然而,复杂食品基质中多种化合物的分析很容易受到基质干扰^[14]^。部分研究尝试采用萃取技术改善基质效应^[15⇓⇓⇓⇓-20]^,但操作耗时,且缺乏基质特异性验证的信息及探讨^[21⇓⇓-24]^。

本研究针对老年人近年来关注的功能性乳粉,将QuEChERS与基于SWATH DIA的超高效液相色谱-四极杆飞行时间质谱(ultra-high performance liquid chromatography-quadrupole-time of flight-mass spectrometry, UHPLC-QTOF-MS)分析技术相结合,对我国相关标准涉及的检测指标补充完善,建立了300种非法添加药物筛查数据库及定量分析方法,检测目标物涉及降血压、降血糖、减肥降脂、镇定安眠及解热镇痛等药物。该方法操作简便,单次进样,无需标准品,即可预判多类功效产品中已知药物的非法添加情况,适用于大批量样品的快速测定。此外,基于EAD碎裂技术获得的丰富二级碎片质谱裂解信息,本工作构建了对样品中未知非法添加药物的识别鉴定策略。该研究成功应用于市售功能性老年乳粉中非法添加物的侦查,识别出已知及未知风险物质共3种。

## 1 实验部分

### 1.1 仪器、试剂与材料

Exion LC 2.0 超高效液相色谱-Zeno TOF 7600四极杆飞行时间质谱,配备Zeno trap离子阱和EAD碰撞室(美国AB SCIEX公司);离心机CR22GⅢ(日本HITACHI公司);电子天平XP205(瑞士Mettler Toledo公司);纯水仪Milli-Q (美国Millipore公司)。

所有化合物标准品均为标准溶液,质量浓度为1 mg/mL,购自天津阿尔塔科技有限公司。甲醇(MeOH)、乙腈(ACN)、甲酸铵(质谱纯,NH_4_HCO_2_)均购于美国ThermoFisher公司;甲酸(质谱纯,FA)购于美国Fluka公司;实验用水为超纯水(符合GB/T 6682-2008一级水要求);硫酸镁(MgSO_4_)、硫酸钠(Na_2_SO_4_)、乙酸钠(NaAc)、氯化钠(NaCl)、柠檬酸(C_6_H_8_O_7_·H_2_O)、十二水合磷酸氢二钠(Na_2_HPO_4_·12H_2_O)、乙二胺四乙酸二钠(Na_2_EDTA)均为分析纯,购于国药集团化学试剂有限公司;EMR-Lipid净化材料购于美国Agilent公司。

市售样品(60个)购自北京、广西、山东等地超市或农贸市场,根据不同样品的市场占有率情况,本研究抽取样品的数量如下:声称增强免疫力/缓解体力疲劳类乳粉共33份,声称辅助调节血糖/血压/血脂类乳粉共15份,声称补肾壮阳类乳粉共12份。购买后置于阴凉处常温保存。

### 1.2 溶液配制

Mcllvaine溶液:将1000 mL 0.1 mol/L的柠檬酸溶液与625 mL 0.2 mol/L磷酸氢二钠混合而得; 0.1 mol/L Na_2_EDTA-Mcllvaine溶液:称取60.5 g乙二胺四乙酸二钠于1625 mL Mcllvaine溶液中,溶解并摇匀。

混合标准中间溶液配制:取适量标准溶液,用乙腈稀释,配制成1 mg/L的混合标准溶液,于4 ℃保存。

空白基质提取液:空白样品按照1.3节条件进行前处理,获得空白基质提取液。

基质混合标准溶液的配制:准确吸取适量混合标准中间液,用空白基质提取液逐级稀释成系列质量浓度的基质混合标准溶液。

### 1.3 样品前处理方法

称取2 g乳粉样品于50 mL具塞离心管中,加入10 mL 40 ℃温水,涡旋1 min,使成均匀乳液。加入10 mL 0.1 mol/L Na_2_EDTA-Mcllvaine溶液-乙腈(2∶8, v/v),随后加入2 g Na_2_SO_4_和0.5 g NaCl进行盐析,漩涡30 s,超声10 min, 4 ℃下以10000 r/min离心5 min。移取4 mL上清液置于含500 mg EMR-Lipid的离心管中,涡旋振荡1 min,再离心5 min (10000 r/min, 4 ℃),取1 mL上清液加入l mL水,混匀后待测。

### 1.4 色谱条件

Kinetex F5色谱柱(100 mm×3.0 mm, 2.6 μm);柱温40 ℃;流速为0.5 mL/min;进样量为10 μL。正离子模式MS检测时,流动相为(A) 5 mmol/L甲酸铵水溶液(含0.1%(v/v)甲酸)和(B)甲醇-乙腈(1∶1, v/v),梯度洗脱。梯度洗脱程序:0~1.0 min, 5%B; 1.0~8.0 min, 5%B~45%B; 8.0~17.0 min, 45%B~60%B; 17.0~20.0 min, 60%B~95%B; 20.0~22.0 min, 95%B; 22.0~22.1 min, 95%B~5%B; 22.1~25.0 min, 5%B。负离子模式MS检测时,流动相为(A)水和(B)甲醇-乙腈(1∶1, v/v),梯度洗脱。梯度洗脱程序:0~1.0 min, 5%B; 1.0~8.0 min, 5%B~45%B; 8.0~17.0 min, 45%B~60%B; 17.0~20.0 min, 60%B~95%B; 20.0~22.0 min, 95%B; 22.0~22.1 min, 95%B~5%B; 22.1~25.0 min, 5%B。

### 1.5 质谱条件

离子源:电喷雾离子源,正/负离子模式。离子源温度550 ℃,喷雾电压(IS) 5500 V,离子源气体1 (GS1)压力379.2 kPa(55 psi),离子源气体2 (GS2)压力379.2 kPa(55 psi),气帘气压力241.3 kPa (35 psi)。扫描模式:Zeno SWATH DIA。一级质谱参数:去簇电压(DP)50 V,质量数扫描范围20~1200,累积时间0.1 s。二级质谱参数:质量数扫描范围20~1200,根据*m/z*的实际分布,共拆分成12段可变窗口。使用SCIEX OS进行数据采集和处理。部分参数见表1。

**表 1:** 300种待测物的理化参数、质谱参数及色谱保留时间

Compound	Molecular formula	Adduct ion	lg P	Primary ion (m/z)	Secondary ion (m/z)	t_R_/ min
GW501516 (GW501516)	C_21_H_18_F_3_NO_3_S_2_	[M+H]^+^	6.2014	454.0753	257.0511	19.30
Acarbose (阿卡波糖)	C_25_H_43_NO_18_	[M+H]^+^	-8.5645	646.2553	304.1425	0.88
Phenformin (苯乙双胍)	C_10_H_15_N_5_	[M+H]^+^	-0.1828	206.1400	60.0570	4.65
Pioglitazone (吡格列酮)	C_19_H_20_N_2_O_3_S	[M+H]^+^	3.1596	357.1267	134.0994	8.31
Acetohexamide (醋磺己脲)	C_15_H_20_N_2_O_4_S	[M+H]^+^	2.2098	325.1217	119.0497	9.91
Dapagliflozin (达格列净)	C_21_H_25_ClO_6_	[M+NH_4_]^+^	1.8444	426.1678	135.0824	10.48
Buformin (丁二胍)	C_6_H_15_N_5_	[M+H]^+^	-0.6254	158.1400	60.0557	1.96
Metformin (二甲双胍)	C_4_H_11_N_5_	[M+H]^+^	-1.2438	130.1087	60.0578	0.95
Voglibose (伏格列波糖)	C_10_H_21_NO_7_	[M+H]^+^	-4.4924	268.1391	92.0726	0.81
Glibenclamide (格列本脲)	C_23_H_28_ClN_3_O_5_S	[M+H]^+^	3.6417	494.1511	369.0676	15.06
Glipizide (格列吡嗪)	C_21_H_27_N_5_O_4_S	[M+H]^+^	2.0781	446.1857	103.0546	10.48
Glibornuride (格列波脲)	C_18_H_26_N_2_O_4_S	[M+H]^+^	2.1685	367.1686	152.1437	12.26
Gliquidone (格列喹酮)	C_27_H_33_N_3_O_6_S	[M+H]^+^	3.5185	528.2163	403.1342	17.96
Glimepiride (格列美脲)	C_24_H_34_N_4_O_5_S	[M+H]^+^	3.0740	491.2323	126.0923	15.31
Gliclazide (格列齐特)	C_15_H_21_N_3_O_3_S	[M+H]^+^	1.6298	324.1376	127.1238	11.07
Tolbutamide (甲苯磺丁脲)	C_12_H_18_N_2_O_3_S	[M+H]^+^	1.7830	271.1111	91.0563	9.69
Canagliflozin (卡格列净)	C_24_H_25_FO_5_S	[M+NH_4_]^+^	2.9683	462.1740	147.0842	12.97
Chlopropamide (氯磺丙脲)	C_10_H_13_ClN_2_O_3_S	[M+H]^+^	1.7379	277.0408	111.0000	9.08
Rosiglitazone (罗格列酮)	C_18_H_19_N_3_O_3_S	[M+H]^+^	2.4909	358.1220	135.0940	7.17
Muraglitazar (莫格他唑)	C_29_H_28_N_2_O_7_	[M+H]^+^	4.4285	517.1969	186.0937	18.41
Nateglinide (那格列奈)	C_19_H_27_NO_3_	[M+H]^+^	3.2609	318.2064	166.0870	13.66
Troglitazone (曲格列酮)	C_24_H_27_NO_5_S	[M+H]^+^	4.3743	442.1683	165.0942	17.17
Repaglinide (瑞格列奈)	C_27_H_36_N_2_O_4_	[M+H]^+^	5.2199	453.2748	230.1916	13.92
Tolazamide (妥拉磺脲)	C_14_H_21_N_3_O_3_S	[M+H]^+^	1.7739	312.1376	115.1246	10.05
Vildagliptin (维达列汀)	C_17_H_25_N_3_O_2_	[M+H]^+^	1.1743	304.2020	154.0980	3.95
Sitagliptin (西他列汀)	C_16_H_15_F_6_N_5_O	[M+H]^+^	2.0165	408.1254	235.0803	7.71
2-Hydroxypropylnortadalafil (2-羟丙基去甲他达拉非)	C_24_H_23_N_3_O_5_	[M+H]^+^	2.4404	434.1711	312.1377	9.93
2-Hydroxyethylnortadalafil (2-羟乙基去甲他达拉非)	C_23_H_21_N_3_O_5_	[M+H]^+^	1.5738	420.1554	298.1192	9.39
N-Phenylpropenyltadalafil (N-苯丙烯基他达拉非)	C_30_H_24_N_4_O_4_	[M+H]^+^	4.2807	505.1870	383.1493	16.13
N-Butyltadalafil (N-丁基他达拉非)	C_25_H_25_N_3_O_4_	[M+H]^+^	3.3816	432.1918	310.1629	13.48
N-Desmethylsildenafil (N-去甲基西地那非)	C_21_H_28_N_6_O_4_S	[M+H]^+^	1.2687	461.1966	283.1221	9.48
N-Desethyl-N-methylvardenafil (N-去乙基-N-甲基伐地那非)	C_22_H_30_N_6_O_4_S	[M+H]^+^	1.6803	475.2122	312.1598	9.02
N-Desethylvardenafil (N-去乙基伐地那非)	C_21_H_28_N_6_O_4_S	[M+H]^+^	1.3381	461.1966	312.1594	9.02
N-Desethylacetildenafil (N-去乙基红地那非)	C_23_H_30_N_6_O_3_	[M+H]^+^	1.7627	439.2452	99.0939	8.47
N-Boc-N-desethyl acetildenafil	C_28_H_38_N_6_O_5_	[M+H]^+^	3.4102	539.2976	439.2524	12.14
(N-叔丁氧羰基-N-去乙基红地那非)						
N-Octylnortadalafil (N-辛基去甲他达拉非)	C_29_H_33_N_3_O_4_	[M+H]^+^	4.9420	488.2544	135.0440	19.07
N-Ethyltadalafil (N-乙基他达拉非)	C_23_H_21_N_3_O_4_	[M+H]^+^	2.6014	404.1605	282.1301	10.90
O-Desethylsildenafil (O-去乙基西地那非)	C_20_H_26_N_6_O_4_S	[M+H]^+^	0.9178	447.1809	283.1240	9.94
Avanafil (阿伐那非)	C_23_H_26_ClN_7_O_3_	[M+H]^+^	2.4318	484.1858	375.1292	9.35
Aildenafil (艾地那非)	C_23_H_32_N_6_O_4_S	[M+H]^+^	2.0457	489.2279	99.0951	10.04
Aminotadalafil (氨基他达拉非)	C_21_H_18_N_4_O_4_	[M+H]^+^	1.4552	391.1401	269.1105	9.23
Aminosildenafil (氨基西地那非)	C_18_H_23_N_5_O_4_S	[M+H]^+^	1.7123	406.1544	364.1116	10.27
Piperiacetildenafil (苯噻啶红地那非)	C_24_H_31_N_5_O_3_	[M+H]^+^	3.3434	438.2500	98.1034	9.09
Xanthoanthrafil (苯酰胺那非)	C_19_H_23_N_3_O_6_	[M+H]^+^	2.3347	390.1660	151.0837	10.55
Pyrazole N-desmethylsildenafil (吡唑N-去甲基西地那非)	C_21_H_28_N_6_O_4_S	[M+H]^+^	1.6005	461.1966	100.1012	8.16
Benzylsildenafil (苄西地那非)	C_28_H_34_N_6_O_4_S	[M+H]^+^	3.1813	551.2435	377.1288	12.63
Propoxyphenyl aildenafil (丙氧苯基艾地那非)	C_24_H_34_N_6_O_4_S	[M+H]^+^	2.4358	503.2435	283.1237	10.96
Propoxyphenyl thioaildenafil	C_24_H_34_N_6_O_3_S_2_	[M+H]^+^	3.8051	519.2207	299.0993	15.95
(丙氧苯基硫代艾地那非)						
Propoxyphenyl thiohomosildenafil (丙氧苯基硫代豪莫西地那非)	C_24_H_34_N_6_O_3_S_2_	[M+H]^+^	3.7604	519.2207	299.0961	15.33
Propoxyphenyl thiohydroxyhomosildenafil	C_24_H_34_N_6_O_4_S_2_	[M+H]^+^	2.7328	535.2156	299.0974	14.54
(丙氧苯基硫代羟基豪莫西地那非)						
Propoxyphenyl thiosildenafil (丙氧苯基硫代西地那非)	C_23_H_32_N_6_O_3_S_2_	[M+H]^+^	3.3703	505.2050	299.0985	14.90
Propoxyphenyl hydroxyhomosildenafil	C_24_H_34_N_6_O_5_S	[M+H]^+^	1.3635	519.2384	99.0923	10.15
(丙氧苯基羟基豪莫西地那非)						
Propoxyphenylsildenafil (丙氧苯基西地那非)	C_23_H_32_N_6_O_4_S	[M+H]^+^	2.0010	489.2279	283.1203	10.27
Propoxyphenylisobutyl aildenafil (丙氧苯基异丁基艾地那非)	C_25_H_36_N_6_O_4_S	[M+H]^+^	2.6818	517.2592	297.1375	11.99
Dapoxetine (达泊西汀)	C_21_H_23_NO	[M+H]^+^	4.9116	306.1852	157.0729	11.75
Dimethylacetildenafil (二甲基红地那非)	C_25_H_34_N_6_O_3_	[M+H]^+^	2.5397	467.2765	410.2192	9.13
Dithio-desmethylcarbodenafil (二硫代去甲基卡巴地那非)	C_23_H_30_N_6_OS_2_	[M+H]^+^	3.9670	471.1995	371.1064	13.60
Dithiodesethyl carbodenafil (二硫代去乙基卡巴地那非)	C_22_H_28_N_6_OS_2_	[M+H]^+^	3.6248	457.1839	371.1053	13.19
Vardenafil N-oxide (伐地那非N-氧化物)	C_23_H_32_N_6_O_5_S	[M+H]^+^	2.0830	505.2228	477.1938	9.29
Vardenafil (伐地那非)	C_23_H_32_N_6_O_4_S	[M+H]^+^	2.0704	489.2279	312.1568	9.18
Vardenafil dimer (伐地那非二聚体)	C_38_H_46_N_10_O_8_S_2_	[M+H]^+^	3.4970	835.3014	312.1560	18.54
Vardenafil oxopiperazine (伐地那非哌嗪酮)	C_21_H_26_N_6_O_5_S	[M+H]^+^	0.8647	475.1758	312.1646	9.81
Vardenafil acetyl analogue (伐地那非乙酰基类似物)	C_24_H_31_N_5_O_3_	[M+H]^+^	2.3504	438.2500	98.1010	8.48
Cinnamyldenafil (桂地那非)	C_32_H_38_N_6_O_3_	[M+H]^+^	4.1885	555.3078	117.0762	12.76
Homosildenafil (豪莫西地那非)	C_23_H_32_N_6_O_4_S	[M+H]^+^	2.0010	489.2279	573.0100	9.69
Acetildenafil (红地那非)	C_25_H_34_N_6_O_3_	[M+H]^+^	2.4950	467.2765	297.1369	8.45
Cyclopentynafil (环戊那非)	C_26_H_36_N_6_O_4_S	[M+H]^+^	2.9237	529.2592	151.0891	9.94
Carbodenafil (卡巴地那非)	C_24_H_32_N_6_O_3_	[M+H]^+^	2.4525	453.2609	339.1513	8.20
Thioaildenafil (硫代艾地那非)	C_23_H_32_N_6_O_3_S_2_	[M+H]^+^	3.4150	505.2050	299.0973	14.55
Thiohomosildenafil (硫代豪莫西地那非)	C_23_H_32_N_6_O_3_S_2_	[M+H]^+^	3.3703	505.2050	113.1084	13.93
Thiosildenafil (硫代西地那非)	C_22_H_30_N_6_O_3_S_2_	[M+H]^+^	2.9802	491.1894	58.0672	13.54
Thioquinapiperfil (硫喹哌非)	C_24_H_28_N_6_OS	[M+H]^+^	4.4828	449.2118	204.1462	8.95
Chlorodenafil (氯地那非)	C_19_H_21_ClN_4_O_3_	[M+H]^+^	3.0963	389.1375	361.1055	13.41
Lodenafil carbonate (罗地那非碳酸酯)	C_47_H_62_N_12_O_11_S_2_	[M+H]^+^	3.1290	1035.4175	487.2118	19.29
Mirodenafil (米罗那非)	C_26_H_37_N_5_O_5_S	[M+H]^+^	2.4514	532.2588	296.1419	12.05
Acetil acid (那非乙酰酸)	C_18_H_20_N_4_O_4_	[M+H]^+^	2.3730	357.1557	329.1290	10.58
Noracetildenafil (那红地那非)	C_24_H_32_N_6_O_3_	[M+H]^+^	2.1049	453.2609	97.0782	8.61
Norneovardenafil (那莫伐地那非)	C_18_H_20_N_4_O_4_	[M+H]^+^	2.4424	357.1557	151.0935	9.64
Norneosildenafil (那莫西地那非)	C_22_H_29_N_5_O_4_S	[M+H]^+^	2.8494	460.2013	283.1222	16.22
Piperazonifil (哌唑那非)	C_25_H_34_N_6_O_4_	[M+H]^+^	1.8723	483.2714	465.2679	8.84
Hydroxyvardenafil (羟基伐地那非)	C_23_H_32_N_6_O_5_S	[M+H]^+^	1.0428	505.2228	312.1585	8.96
Hydroxyhomosildenafil (羟基豪莫西地那非)	C_23_H_32_N_6_O_5_S	[M+H]^+^	0.9734	505.2228	487.2150	9.40
Hydroxyacetildenafil (羟基红地那非)	C_25_H_34_N_6_O_4_	[M+H]^+^	1.4674	483.2714	127.0892	8.45
Hydroxythiovardenafil (羟基硫代伐地那非)	C_23_H_32_N_6_O_4_S_2_	[M+H]^+^	2.4121	521.1999	328.1368	11.54
Hydroxythiohomosildenafil (羟基硫代豪莫西地那非)	C_23_H_32_N_6_O_4_S_2_	[M+H]^+^	2.3427	521.1999	99.0943	13.31
Hydroxythioacetildenafil (羟基硫代红地那非)	C_25_H_34_N_6_O_3_S	[M+H]^+^	2.8367	499.2486	127.0869	11.94
Hydroxychlorodenafil (羟基氯地那非)	C_19_H_23_ClN_4_O_3_	[M+H]^+^	2.9470	391.1531	313.1372	11.78
Gendenafil (庆地那非)	C_19_H_22_N_4_O_3_	[M+H]^+^	2.8774	355.1765	327.1490	12.16
Desmethylcarbodenafil (去甲基卡巴地那非)	C_23_H_30_N_6_O_3_	[M+H]^+^	2.0624	439.2452	339.1533	7.97
Desmethylthiosildenafil (去甲基硫代西地那非)	C_21_H_28_N_6_O_3_S_2_	[M+H]^+^	2.6380	477.1737	85.0767	13.40
Demethylpiperaziny sildenafil sulfonic acid	C_17_H_20_N_4_O_5_S	[M+H]^+^	1.9215	393.1227	365.0956	7.28
(去甲基哌嗪基西地那非磺酸)						
Nortadalafil (去甲基他达拉非)	C_21_H_17_N_3_O_4_	[M+H]^+^	1.8691	376.1292	254.0993	9.39
Descarbonsildenafil (去碳西地那非)	C_21_H_30_N_6_O_4_S	[M+H]^+^	1.5147	463.2122	418.1562	8.82
Desethylcarbodenafil (去乙基卡巴地那非)	C_22_H_28_N_6_O_3_	[M+H]^+^	1.7202	425.2296	311.1200	8.20
Dichlorodenafil (双氯地那非)	C_19_H_20_Cl_2_N_4_O_2_	[M+H]^+^	4.4508	407.1036	350.0375	19.15
Didescarbonsildenafil (双去碳西地那非)	C_20_H_28_N_6_O_4_S	[M+H]^+^	1.1725	449.1966	311.1530	8.63
Dioxohongdenafil (双酮红地那非)	C_25_H_30_N_6_O_5_	[M+H]^+^	1.5482	495.2350	127.0908	10.15
Tadalafil (他达拉非)	C_22_H_19_N_3_O_4_	[M+H]^+^	2.2113	390.1448	268.1138	10.10
Tadalafil dichloro impurity (他达拉非二氯代杂质)	C_22_H_18_Cl_2_N_2_O_5_	[M+H]^+^	3.7160	461.0666	274.0868	15.96
Chloropretadalafil (他达拉非甲基氯化物)	C_22_H_19_ClN_2_O_5_	[M+H]^+^	3.1511	427.1055	135.0457	13.73
Oxohongdenafil (酮红地那非)	C_25_H_32_N_6_O_4_	[M+H]^+^	2.0216	481.2558	410.2216	10.36
Imidazosagatriazinone (脱硫伐地那非)	C_17_H_20_N_4_O_2_	[M+H]^+^	2.6748	313.1659	285.1431	13.89
Depiperazinothiosildenafil (脱哌嗪基硫代西地那非)	C_17_H_20_N_4_O_4_S_2_	[M+H]^+^	3.2908	409.0999	381.0706	9.69
Pseudovardenafil (伪伐地那非)	C_22_H_29_N_5_O_4_S	[M+H]^+^	2.9188	460.2013	312.1604	14.51
Udenafil (乌地那非)	C_25_H_36_N_6_O_4_S	[M+H]^+^	2.8275	517.2592	283.1220	10.13
Sildenafil N-oxide (西地那非N-氧化物)	C_22_H_30_N_6_O_5_S	[M+H]^+^	1.6235	491.2071	404.1414	9.71
Sildenafil (西地那非)	C_22_H_30_N_6_O_4_S	[M+H]^+^	1.6109	475.2122	283.1205	9.48
Sildenafil dimer impurity (西地那非二聚体杂质)	C_38_H_46_N_10_O_8_S_2_	[M+H]^+^	3.4970	835.3014	311.1501	19.49
Sildenafil impurity 12 (西地那非杂质12)	C_25_H_34_N_6_OS_2_	[M+H]^+^	3.4698	499.2308	468.1891	14.78
Sildenafil impurity 14 (西地那非杂质14)	C_24_H_32_N_6_OS_2_	[M+H]^+^	4.4018	485.2152	371.1051	14.53
Nitrodenafil (硝地那非)	C_17_H_19_N_5_O_4_	[M+H]^+^	2.5830	358.1510	330.1243	14.03
Mutaprodenafil (亚硝地那非)	C_27_H_35_N_9_O_5_S_2_	[M+H]^+^	3.5453	630.2275	142.0094	12.12
Acetaminotadalafil (乙酰胺基他达拉非)	C_23_H_20_N_4_O_5_	[M+H]^+^	1.6326	433.1507	262.0870	9.23
Acetylvardenafil (乙酰伐地那非)	C_25_H_34_N_6_O_3_	[M+H]^+^	2.5644	467.2765	111.0927	8.84
Isobutylsildenafil (异丁基西地那非)	C_23_H_32_N_6_O_4_S	[M+H]^+^	1.8569	489.2279	58.0647	10.28
Yohimbine (育亨宾)	C_21_H_26_N_2_O_3_	[M+H]^+^	2.6471	355.2016	212.1315	7.23
N,N-Didesmethyl sibutramine (N,N-双去甲基西布曲明)	C_15_H_22_ClN	[M+H]^+^	4.1351	252.1514	125.0233	11.02
N-Monodesmethyl sibutramine (N-单去甲基西布曲明)	C_16_H_24_ClN	[M+H]^+^	4.3958	266.1670	125.0229	11.19
Alprazolam (阿普唑仑)	C_17_H_13_ClN_4_	[M+H]^+^	3.5801	309.0902	281.0793	9.91
Atenolol (阿替洛尔)	C_14_H_22_N_2_O_3_	[M+H]^+^	0.4521	267.1703	145.0668	3.63
Estazolam (艾司唑仑)	C_16_H_11_ClN_4_	[M+H]^+^	3.2717	295.0745	267.0630	9.32
Tranexamic acid (氨甲环酸)	C_8_H_15_NO_2_	[M+H]^+^	0.8361	158.1176	140.1075	1.55
Amlodipine (氨氯地平)	C_20_H_25_ClN_2_O_5_	[M+H]^+^	2.2663	409.1525	238.0690	10.52
Oxazepam (奥沙西泮)	C_15_H_11_ClN_2_O_2_	[M+H]^+^	2.4479	287.0582	241.0616	9.09
Aceclofenac (醋氯芬酸)	C_16_H_13_Cl_2_NO_4_	[M+H]^+^	3.9073	354.0294	214.0510	12.91
Diazepam (地西泮)	C_16_H_13_ClN_2_O	[M+H]^+^	3.1538	285.0789	193.0967	11.05
Dioxopromethazine (二氧丙嗪)	C_17_H_20_N_2_O_2_S	[M+H]^+^	2.9210	317.1318	167.0790	7.48
Felodipine (非洛地平)	C_18_H_19_Cl_2_NO_4_	[M+H]^+^	3.9643	384.0764	338.0405	15.65
Fenfluramine (芬氟拉明)	C_12_H_16_F_3_N	[M+H]^+^	3.2459	232.1308	159.0440	8.40
Phenolphthalein (酚酞)	C_20_H_14_O_4_	[M+H]^+^	3.5601	319.0965	225.0633	9.35
Captopril (卡托普利)	C_9_H_15_NO_3_S	[M+H]^+^	-0.7068	218.0845	70.0714	5.05
Clonidine (可乐定)	C_9_H_9_Cl_2_N_3_	[M+H]^+^	2.3645	230.0246	212.9986	4.22
Lorazepam (劳拉西泮)	C_15_H_10_Cl_2_N_2_O_2_	[M+H]^+^	3.1013	321.0192	275.0224	9.23
Reserpine (利血平)	C_33_H_40_N_2_O_9_	[M+H]^+^	4.1711	609.2807	195.0724	13.02
Chlorphenamine (氯苯那敏)	C_16_H_19_ClN_2_	[M+H]^+^	3.8186	275.1310	230.0796	7.97
Chlordiazepoxide (氯氮卓)	C_16_H_14_ClN_3_O	[M+H]^+^	2.9507	300.0898	227.0578	7.64
Chlormezanone (氯美扎酮)	C_11_H_12_ClNO_3_S	[M+H]^+^	1.6155	274.0299	154.0432	7.87
Clonazepam (氯硝西泮)	C_15_H_10_ClN_3_O_3_	[M+H]^+^	3.0377	316.0484	270.0635	9.53
Tetrahydropalmatine (罗通定)	C_21_H_25_NO_4_	[M+H]^+^	3.3765	356.1856	192.1083	7.99
Lovastatin (洛伐他汀)	C_24_H_36_O_5_	[M+H]^+^	4.1955	405.2636	199.1521	17.37
Lovastatin hydroxy acid, sodium salt (洛伐他汀羟酸钠盐)	C_24_H_37_NaO_6_	[M+H]^+^	2.3809	445.2561	343.1867	15.53
Ephedrine (麻黄碱)	C_10_H_15_NO	[M+H]^+^	1.3279	166.1226	133.0922	3.59
Mevastatin (美伐他汀)	C_23_H_34_O_5_	[M+H]^+^	3.9495	391.2479	159.1201	15.93
Midazolam (咪达唑仑)	C_18_H_13_ClFN_3_	[M+H]^+^	4.3242	326.0855	291.1225	8.69
Nimodipine (尼莫地平)	C_21_H_26_N_2_O_7_	[M+H]^+^	2.9708	419.1813	301.0902	14.68
Nitrendipine (尼群地平)	C_18_H_20_N_2_O_6_	[M+H]^+^	2.5657	361.1394	315.1040	13.55
Nisoldipine (尼索地平)	C_20_H_24_N_2_O_6_	[M+H]^+^	3.2018	389.1707	239.0820	15.18
Prazosin (哌唑嗪)	C_19_H_21_N_5_O_4_	[M+H]^+^	1.7846	384.1666	247.1269	7.59
Sinomenine (青藤碱)	C_19_H_23_NO_4_	[M+H]^+^	2.0181	330.1700	181.0680	4.64
Triazolam (三唑仑)	C_17_H_12_Cl_2_N_4_	[M+H]^+^	4.2335	343.0512	308.0888	9.95
Salbutamol (沙丁胺醇)	C_13_H_21_NO_3_	[M+H]^+^	1.3060	240.1594	148.0835	3.45
Melatonine (褪黑素)	C_13_H_16_N_2_O_2_	[M+H]^+^	1.8551	233.1285	174.0987	7.26
Dehydro lovastatin (脱羟基洛伐他丁)	C_24_H_34_O_4_	[M+H]^+^	5.0007	387.2530	199.1560	19.32
Venlafaxine (文拉法辛)	C_17_H_27_NO_2_	[M+H]^+^	3.0356	278.2115	58.0712	7.48
Sibutramine (西布曲明)	C_17_H_26_ClN	[M+H]^+^	4.7380	280.1827	125.0228	11.47
Nifedipine (硝苯地平)	C_17_H_18_N_2_O_6_	[M+H]^+^	2.1756	345.1081	254.1047	11.19
Nitrazepam (硝西泮)	C_15_H_11_N_3_O_3_	[M+H]^+^	2.3843	282.0873	236.1016	9.26
Simvastatin (辛伐他汀)	C_25_H_38_O_5_	[M+H]^+^	4.5856	419.2792	199.1494	18.64
Nicotinic acid (烟酸)	C_6_H_5_NO_2_	[M+H]^+^	0.7798	124.0393	80.0531	1.15
Zaleplon (扎来普隆)	C_17_H_15_N_5_O	[M+H]^+^	2.6408	306.1349	236.1009	9.11
Zopiclone (佐匹克隆)	C_17_H_17_ClN_6_O_3_	[M+H]^+^	1.5680	389.1123	245.0290	6.49
Phentolamine (酚妥拉明)	C_17_H_19_N_3_O	[M+H]^+^	2.8404	282.1601	212.1130	7.56
Terazosin (特拉唑嗪)	C_19_H_25_N_5_O_4_	[M+H]^+^	1.0568	388.1979	290.1652	6.80
Tolazoline (妥拉唑林)	C_10_H_12_N_2_	[M+H]^+^	1.2308	161.1073	91.0593	3.35
Chrysophanic acid (大黄酚)	C_15_H_10_O_4_	[M+H]^+^	2.1816	255.0652	181.0644	17.15
Glabridin (橙黄决明素)	C_17_H_14_O_7_	[M+H]^+^	1.9044	331.0812	270.0565	11.39
Sodium picosulfate (匹可硫酸钠)	3C_18_H_13_NO_8_S_2_H_2_	[M+H]^+^	1.9398	438.0312	184.0760	11.43
Amphetamine (安非他明)	C_9_H_13_N	[M+H]^+^	1.5763	136.1121	91.0595	4.25
Bupropion (安非他酮)	C_13_H_18_ClNO	[M+H]^+^	3.2993	240.1150	131.0792	7.67
Orlistat (奥利司他)	C_29_H_53_NO_5_	[M+H]^+^	6.8819	496.3997	319.2993	20.66
Norephedrine (苯丙醇胺)	C_9_H_13_NO	[M+H]^+^	1.0672	152.1070	134.0968	2.75
Bezafibrate (苯扎贝特)	C_19_H_20_ClNO_4_	[M+H]^+^	3.5545	362.1154	138.9999	11.26
Bisacodyl (比沙可啶)	C_22_H_19_NO_4_	[M+H]^+^	4.1124	362.1387	184.0816	11.44
11-Desisobutyl-11-benzyl sibutramine (苄基西布曲明)	C_20_H_24_ClN	[M+H]^+^	4.9346	314.1670	91.0607	12.21
Bumetanide (布美他尼)	C_17_H_20_N_2_O_5_S	[M+H]^+^	3.0365	365.1166	240.1418	10.86
Fenofibrate (非诺贝特)	C_20_H_21_ClO_4_	[M+H]^+^	4.6800	361.1201	233.0368	19.34
Phentermine (分特拉明)	C_10_H_15_N	[M+H]^+^	1.9664	150.1277	91.0601	5.12
Fluoxetine (氟西汀)	C_17_H_18_F_3_NO	[M+H]^+^	4.4350	310.1413	44.0546	11.58
Homosibutramine (豪莫西布曲明)	C_18_H_28_ClN	[M+H]^+^	5.1281	294.1983	125.0219	12.23
Methamphetamine (甲基安非他明)	C_10_H_15_N	[M+H]^+^	1.8370	150.1277	91.0601	4.78
Methylephedrine (甲基麻黄碱)	C_11_H_17_NO	[M+H]^+^	1.6701	180.1383	162.1310	4.07
Caffeine (咖啡因)	C_8_H_10_N_4_O_2_	[M+H]^+^	-1.0293	195.0877	138.0659	4.79
Rimonabant (利莫那班)	C_22_H_21_Cl_3_N_4_O	[M+H]^+^	5.9386	463.0854	362.9922	18.82
Chloro sibutramine (氯代西布曲明)	C_17_H_25_Cl_2_N	[M+H]^+^	5.3914	314.1437	158.9829	13.07
Lorcaserin (氯卡色林)	C_11_H_14_ClN	[M+H]^+^	2.5892	196.0888	129.0744	7.18
Cathine (去甲伪麻黄碱)	C_9_H_13_NO	[M+H]^+^	1.0672	152.1070	134.0987	3.00
Pseudoephedrine (伪麻黄碱)	C_10_H_15_NO	[M+H]^+^	1.3279	166.1226	115.0560	3.59
Indapamide (吲达帕胺)	C_16_H_16_ClN_3_O_3_S	[M+H]^+^	2.0834	366.0674	132.0857	8.88
Dehydro nifedipin (去氢硝苯地平)	C_17_H_16_N_2_O_6_	[M+H]^+^	2.8468	345.1081	284.0963	10.53
Dehydronitroso nifedipin (去氢亚硝基硝苯地平)	C_17_H_16_N_2_O_5_	[M+H]^+^	3.3365	329.1132	284.0989	10.83
Higenamine (去甲乌药碱)	C_16_H_17_NO_3_	[M+H]^+^	2.2329	272.1281	107.0502	4.34
Tretoquinol (曲托喹酚)	C_19_H_23_NO_5_	[M+H]^+^	2.5531	346.1649	164.0734	5.97
Octopamin (奥克巴胺)	C_8_H_11_NO_2_	[M+H]^+^	0.3843	136.0757	119.0510	1.01
Morphine (吗啡)	C_17_H_19_NO_3_	[M+H]^+^	1.1981	286.1438	165.0714	2.50
Codeine (可待因)	C_18_H_21_NO_3_	[M+H]^+^	1.5011	300.1594	165.0713	4.25
Papaverine (罂粟碱)	C_20_H_21_NO_4_	[M+H]^+^	3.8600	340.1543	202.0912	7.90
Noscapine (那可丁)	C_22_H_23_NO_7_	[M+H]^+^	2.8818	414.1547	220.1027	7.89
Thebaine (蒂巴因)	C_19_H_21_NO_3_	[M+H]^+^	2.4245	312.1594	58.0700	6.61
Meloxicam (美洛昔康)	C_14_H_13_N_3_O_4_S_2_	[M+H]^+^	1.9509	352.0420	115.0339	10.70
Sulindac (舒林酸)	C_20_H_17_FO_3_S	[M+H]^+^	4.3655	357.0955	233.0769	11.44
Piroxicam (吡罗昔康)	C_15_H_13_N_3_O_4_S	[M+H]^+^	1.5810	332.0700	95.0664	9.17
Benorilate (贝诺酯)	C_17_H_15_NO_5_	[M+H]^+^	2.7895	314.1023	121.0293	9.85
Etoricoxib (依托考昔)	C_18_H_15_ClN_2_O_2_S	[M+H]^+^	4.1759	359.0616	280.0805	8.84
Naproxen (萘普生)	C_14_H_14_O_3_	[M+H]^+^	3.0365	231.1016	185.0999	11.22
Fenbufen (芬布芬)	C_16_H_14_O_3_	[M+H]^+^	3.4011	255.1016	105.0340	10.54
Oxaprozin (奥沙普秦)	C_18_H_15_NO_3_	[M+H]^+^	4.0258	294.1125	103.0565	12.42
Nabumetone (萘丁美酮)	C_15_H_16_O_2_	[M+H]^+^	3.3700	229.1223	171.0819	13.22
Feprazone (非普拉宗)	C_20_H_20_N_2_O_2_	[M+H]^+^	3.9539	321.1598	253.0984	13.18
Celecoxib (塞来昔布)	C_17_H_14_F_3_N_3_O_2_S	[M+H]^+^	3.5139	382.0832	362.0765	15.36
Alclomethasonedipropionate (阿氯米松双丙酸酯)	C_28_H_37_ClO_7_	[M+H]^+^	2.0209	521.2301	301.1584	15.89
Antipyrine (安替比林)	C_11_H_12_N_2_O	[M+H]^+^	1.4844	189.1022	56.0517	5.62
Amcinonide (安西奈德)	C_28_H_35_FO_7_	[M+H]^+^	3.5238	503.2440	99.0917	15.96
Aminophenazone (氨基比林)	C_13_H_17_N_3_O	[M+H]^+^	1.5504	232.1444	97.0814	4.09
Phenylbutazone (保泰松)	C_19_H_20_N_2_O_2_	[M+H]^+^	3.7878	395.1864	357.1707	7.48
Beclomethasone (倍氯米松)	C_22_H_29_ClO_5_	[M+H]^+^	2.1650	409.1776	391.1669	9.36
Beclometasonedipropionate (倍氯米松双丙酸酯)	C_28_H_37_ClO_7_	[M+H]^+^	4.0868	521.2301	57.0339	18.07
Betamethasone (倍他米松)	C_22_H_29_FO_5_	[M+H]^+^	1.8957	393.2072	147.0799	9.36
Betamethasone 21-acetate (倍他米松醋酸酯)	C_24_H_31_FO_6_	[M+H]^+^	2.4665	435.2177	147.0802	11.65
Betamethasone dipropionate (倍他米松双丙酸酯)	C_28_H_37_FO_7_	[M+H]^+^	3.8175	505.2596	279.1732	17.21
Betamethasone 17-valerate (倍他米松戊酸酯)	C_27_H_37_FO_6_	[M+H]^+^	3.6368	477.2647	279.1739	15.93
Budesonide (布地奈德)	C_25_H_34_O_6_	[M+H]^+^	2.7168	431.2428	147.0809	12.07
Deflazacort (地夫可特)	C_25_H_31_NO_6_	[M+H]^+^	2.5632	442.2224	424.2131	11.29
Dexamethasone (地塞米松)	C_22_H_29_FO_5_	[M+H]^+^	1.8957	393.2072	147.0827	9.36
Dexamethasone 21-acetate (地塞米松醋酸酯)	C_24_H_31_FO_6_	[M+H]^+^	2.4665	435.2177	147.0801	11.65
Acetaminophen (对乙酰氨基酚)	C_8_H_9_NO_2_	[M+H]^+^	1.3506	152.0706	110.0657	7.01
Diflorasonediacetate (二氟拉松双醋酸酯)	C_26_H_32_F_2_O_7_	[M+H]^+^	2.9853	495.2189	43.0182	14.98
Phenacetin (非那西丁)	C_10_H_13_NO_2_	[M+H]^+^	2.0437	180.1019	110.0635	7.01
Fluoromethalone (氟米龙)	C_22_H_29_FO_4_	[M+H]^+^	2.9233	377.2123	279.1740	10.18
Fluorometholone 17-acetate (氟米龙醋酸酯)	C_24_H_31_FO_5_	[M+H]^+^	3.4941	419.2228	279.1772	12.60
Flumethasone (氟米松)	C_22_H_28_F_2_O_5_	[M+H]^+^	1.8437	411.1978	121.0648	9.62
Fludrocortisone 21-acetate (氟氢可的松醋酸酯)	C_23_H_31_FO_6_	[M+H]^+^	2.4445	423.2177	213.1274	10.29
Fludroxycortide (氟氢缩松)	C_24_H_33_FO_6_	[M+H]^+^	2.4987	437.2334	181.1022	10.27
Fluocinonide (氟轻松醋酸酯)	C_26_H_32_F_2_O_7_	[M+H]^+^	2.9376	495.2189	121.0648	14.47
Fluticasone propionate (氟替卡松丙酸酯)	C_25_H_31_F_3_O_5_S	[M+H]^+^	4.4300	501.1917	293.1542	17.22
Prednicarbate (哈西奈德)	C_27_H_36_O_8_	[M+H]^+^	3.6993	489.2483	57.0336	15.35
Sulfamethoxazole (磺胺甲恶唑)	C_10_H_11_N_3_O_3_S	[M+H]^+^	1.3660	254.0594	108.0449	6.58
Mefenamic acid (甲芬那酸)	C_15_H_15_NO_2_	[M+H]^+^	3.7452	242.1176	224.1075	15.41
Methylprednisolone (甲基泼尼松龙)	C_22_H_30_O_5_	[M+H]^+^	1.8036	375.2166	161.0967	9.19
Methylprednisolone 21-acetate (甲基泼尼松龙醋酸酯)	C_24_H_32_O_6_	[M+H]^+^	2.3744	417.2272	253.1596	11.26
Trimethoprim (甲氧苄啶)	C_14_H_18_N_4_O_3_	[M+H]^+^	1.2576	291.1452	123.0690	5.28
Cortisone (可的松)	C_21_H_28_O_5_	[M+H]^+^	1.9898	361.2010	163.1113	8.55
Cortisone 21-acetate (可的松醋酸酯)	C_23_H_30_O_6_	[M+H]^+^	2.5606	403.2115	163.1113	10.83
Clobetasone 17-butyrate (氯倍他松丁酸酯)	C_26_H_32_ClFO_5_	[M+H]^+^	4.7014	479.1995	279.1384	18.78
Clobetasol 17-propionate (氯倍他索丙酸酯)	C_25_H_32_ClFO_5_	[M+H]^+^	4.1031	467.1995	355.1446	15.95
Mometasonefuroate (莫米他松糠酸酯)	C_27_H_30_Cl_2_O_6_	[M+H]^+^	4.8692	521.1492	95.0128	16.69
Halcinonide (泼尼卡酯)	C_24_H_32_ClFO_5_	[M+H]^+^	3.8893	455.1995	279.1739	15.08
Prednisone (泼尼松)	C_21_H_26_O_5_	[M+H]^+^	1.7658	359.1853	341.1756	8.47
Prednisone 21-acetate (泼尼松醋酸酯)	C_23_H_28_O_6_	[M+H]^+^	1.7658	401.1959	147.0806	10.76
Prednisolone (泼尼松龙)	C_21_H_28_O_5_	[M+H]^+^	1.5576	361.2010	147.0807	8.40
Prednisolone-21-acetate (泼尼松龙醋酸酯)	C_23_H_30_O_6_	[M+H]^+^	2.1284	403.2115	385.2032	10.16
Hydrocortisone (氢化可的松)	C_21_H_30_O_5_	[M+H]^+^	1.7816	363.2166	121.0694	8.41
Hydrocortisone acetate (氢化可的松醋酸酯)	C_23_H_32_O_6_	[M+H]^+^	2.3524	405.2272	309.1853	10.22
Hydrocortisone-17-butyrate (氢化可的松丁酸酯)	C_25_H_36_O_6_	[M+H]^+^	3.1326	433.2585	121.0653	12.08
Hydrocortisone 17-valerate (氢化可的松戊酸酯)	C_26_H_38_O_6_	[M+H]^+^	3.5227	447.2741	121.0656	13.51
Tramcinoloneacetonide (曲安奈德)	C_24_H_31_FO_6_	[M+H]^+^	2.4188	435.2177	213.1280	10.27
Triamcinolone acetonide acetate (曲安奈德醋酸酯)	C_26_H_33_FO_7_	[M+H]^+^	2.9896	477.2283	213.1267	13.79
Triamcinolone (曲安西龙)	C_21_H_27_FO_6_	[M+H]^+^	0.6205	395.1864	371.1543	7.48
Triamcinolone diacetate (曲安西龙双醋酸酯)	C_25_H_31_FO_8_	[M+H]^+^	1.7621	479.2076	321.1489	10.25
Ketoprofen (酮洛芬)	C_16_H_14_O_3_	[M+H]^+^	3.1057	255.1016	105.0340	10.54
Diphenhydramine hydrochloride (盐酸苯海拉明)	C_17_H_21_NO	[M+H]^+^	3.3542	256.1696	167.0917	8.80
Propyphenazone (异丙安替比林)	C_14_H_18_N_2_O	[M+H]^+^	2.6078	231.1492	189.1052	8.80
Atropine sulfate (阿托品)	C_17_H_23_NO_3_	[M+H]^+^	1.9309	290.1751	124.1155	5.57
Anisodamine (山莨菪碱)	C_17_H_23_NO_4_	[M+H]^+^	0.9017	306.1700	140.1085	4.36
Scopolamine hydrobromide (东莨菪碱)	C_17_H_21_NO_4_	[M+H]^+^	0.9181	304.1543	138.0941	4.61
Procaine (普鲁卡因)	C_13_H_20_N_2_O_2_	[M+H]^+^	1.7674	120.0480		4.52
Lidocaine (利多卡因)	C_14_H_22_N_2_O	[M+H]^+^	2.5837	235.1805	86.1013	5.59
Norfloxacin (诺氟沙星)	C_16_H_18_FN_3_O_3_	[M+H]^+^	1.2683	320.1405	302.1316	6.25
Ofloxacin (氧氟沙星)	C_18_H_20_FN_3_O_4_	[M+H]^+^	1.5440	362.1511	261.1055	6.23
Lomefloxacin (洛美沙星)	C_17_H_19_F_2_N_3_O_3_	[M+H]^+^	1.7959	352.1467	265.1155	6.62
Pefloxacin (培氟沙星)	C_17_H_20_FN_3_O_3_	[M+H]^+^	1.6105	334.1561	316.1476	6.33
Fleroxacin (氟罗沙星)	C_17_H_18_F_3_N_3_O_3_	[M+H]^+^	1.6992	370.1373	352.1291	6.13
Sarafloxacin (沙拉沙星)	C_20_H_17_F_2_N_3_O_3_	[M+H]^+^	2.3767	386.1311	368.1218	7.57
Difloxacin (双氟沙星)	C_21_H_19_F_2_N_3_O_3_	[M+H]^+^	2.7189	400.1467	382.1388	7.63
Sparfloxacin (司帕沙星)	C_19_H_22_F_2_N_4_O_3_	[M+H]^+^	2.0816	393.1733	349.1854	7.73
Ciprofloxacin (环丙沙星)	C_17_H_18_FN_3_O_3_	[M+H]^+^	1.5833	332.1405	314.1309	6.44
Danofloxacinmesylate (达氟沙星)	C_19_H_20_FN_3_O_3_	[M+H]^+^	2.0664	358.1561	340.1492	6.56
Enrofloxacin (恩诺沙星)	C_19_H_22_FN_3_O_3_	[M+H]^+^	2.3156	360.1718	342.1655	6.84
Ciglitazone (环格列酮)	C_18_H_23_NO_3_S	[M-H]^-^	3.9299	332.1326	150.0139	18.72
Furosemide (呋塞米)	C_12_H_11_ClN_2_O_5_S	[M-H]^-^	1.8907	329.0004	204.9939	6.20
Hydrochlorothiazide (氢氯噻嗪)	C_7_H_8_ClN_3_O_4_S_2_	[M-H]^-^	-0.3513	295.9572	77.9697	4.40
Secobarbital (司可巴比妥)	C_12_H_18_N_2_O_3_	[M-H]^-^	1.3511	237.1245	194.1188	8.93
Phenobarbital (苯巴比妥)	C_12_H_12_N_2_O_3_	[M-H]^-^	0.7004	231.0775	205.0555	6.90
Amobarbital (异戊巴比妥)	C_11_H_18_N_2_O_3_	[M-H]^-^	1.1850	225.1245	182.1205	8.44
Barbital (巴比妥)	C_8_H_12_N_2_O_3_	[M-H]^-^	0.1588	183.0775	140.0722	4.50
Sennoside A (番泻苷A)	C_42_H_38_O_20_	[M-H]^-^	-1.0956	861.1884	386.1051	5.16
Sennoside B (番泻苷B)	C_42_H_38_O_20_	[M-H]^-^	-1.0956	861.1884	386.1038	4.45
Emodin-3-methyl ether (大黄素甲醚)	C_16_H_12_O_5_	[M-H]^-^	2.1902	283.0612	240.0436	19.11
Diflunisal (二氟尼柳)	C_13_H_8_F_2_O_3_	[M-H]^-^	3.0356	249.0369	205.0558	6.95
Nimesulide (尼美舒利)	C_13_H_12_N_2_O_5_S	[M-H]^-^	2.7586	307.0394	229.0612	12.08
Flurbiprofen (氟比洛芬)	C_15_H_13_FO_2_	[M-H]^-^	3.6808	243.0827	101.0400	12.38
Diclofenac sodium (双氯芬酸钠)	C_14_H_10_Cl_2_NNaO_2_	[M-H]^-^	0.0334	296.0065	252.0162	11.81
Etodolac (依托度酸)	C_17_H_21_NO_3_	[M-H]^-^	3.3830	286.1449	212.1443	12.28
Indometacin (吲哚美辛)	C_19_H_16_ClNO_4_	[M-H]^-^	3.9273	356.0695	158.0610	13.84
Chlorzoxazone (氯唑沙宗)	C_7_H_4_ClNO_2_	[M-H]^-^	1.7745	167.9858	132.0113	7.88
Acetylsalicylic acid (阿司匹林)	C_9_H_8_O_4_	[M-H]^-^	1.3101	137.0244	93.0395	2.10
Ibuprofen (布洛芬)	C_13_H_18_O_2_	[M-H]^-^	3.0732	205.1234	143.0871	12.53
Analgin (安乃近)	C_13_H_17_N_3_O_4_S	[M-H]^-^	0.4233	310.0867	175.0397	7.48
Sulbactam (舒巴坦)	C_8_H_11_NO_5_S	[M-H]^-^	-0.7950	232.0285	188.0400	0.78
Chlorothiazide (氯噻嗪)	C_7_H_6_ClN_3_O_4_S_2_	[M-H]^-^	0.1299	293.9415	213.9664	4.11
Pravastatin (普伐他汀)	C_23_H_36_O_7_	[M-H]^-^	2.4404	423.2388	327.1624	8.48

lg *P*: calculated by built-in function of ChemDraw.

### 1.6 数据库的建立及使用

将质量浓度为100 μg/L的单一分析物标准溶液注入UHPLC-QTOF-MS中,并进行数据采集,以低、中、高碰撞能量(20、35、50 eV)的DIA扫描模式,利用动态背景过滤(DBS)功能自动扣除背景离子,建立了包含精确质量数和片段离子的MS/MS谱库。筛查确认原则设置:精确质量数偏差绝对值≤5×10^-6^(5 ppm),保留时间误差≤0.2 min,差异同位素比≤10%,检索匹配分值≥90。

### 1.7 未知结构类似物的确证

取待测样品,按照1.3节方法处理,采用优化后的色谱条件进行分离,确定保留时间后,按照优化后的质谱条件进行一级质谱扫描,得到准分子离子峰,经拟合确定化合物的分子式;对母离子进行子离子扫描,得到二级碎片质谱图,进而推断化合物母核及丢失碎片等基本结构。

## 2 结果与讨论

### 2.1 色谱条件的优化

#### 2.1.1 色谱柱的选择

目标分析物种类广泛,理化性质各异,涉及多组精确质量数相似或同分异构体化合物。为了最大限度地兼顾所有组分的分离度和响应值,需要对色谱和质谱条件进行逐一优化,从而实现300种化合物的同步UHPLC-Q-TOF/MS分析。一般来说,保留和分离的机制可能包括疏水相互作用、离子交换、离子对、表面局部化等。对于某些含有不饱和结构或平面结构的化合物,另一个重要的选择性因素是*π*-*π*相互作用。本研究采用Kinetex五氟苯基柱实现了较好的分离效果。

#### 2.1.2 流动相的选择

在色谱条件的优化过程中,考虑到非法添加化学药物中有多组同分异构体,如吡唑*N*-去甲基西地那非、*N*-去乙基伐地那非和*N*-去甲基西地那非3种化合物的质荷比在正离子模式下均为461.1966,即使高分辨质谱也无法区分,有必要通过色谱条件的优化实现有效分离。如图1a~e所示,当甲醇作为流动相的有机相、并在水相中添加2.5、5.0、10.0 mmol/L甲酸铵时,采用1.4节下梯度洗脱程序进行分析,这3种化合物的色谱保留均较强,色谱分离度相对较好,但色谱峰形较差,特别是*N*-去甲基西地那非,色谱峰出现不同程度的分叉;当有机相更换为乙腈时,3种化合物均得到了比较好的色谱峰形,但分离度和色谱保留效果不及甲醇,故最终采用甲醇-乙腈(1∶1, v/v)作为流动相的有机相,实现了色谱峰形、分离度和保留行为的同时兼顾。在优化的色谱条件下,上述3种同分异构体得到了良好的分离。

**图1:**
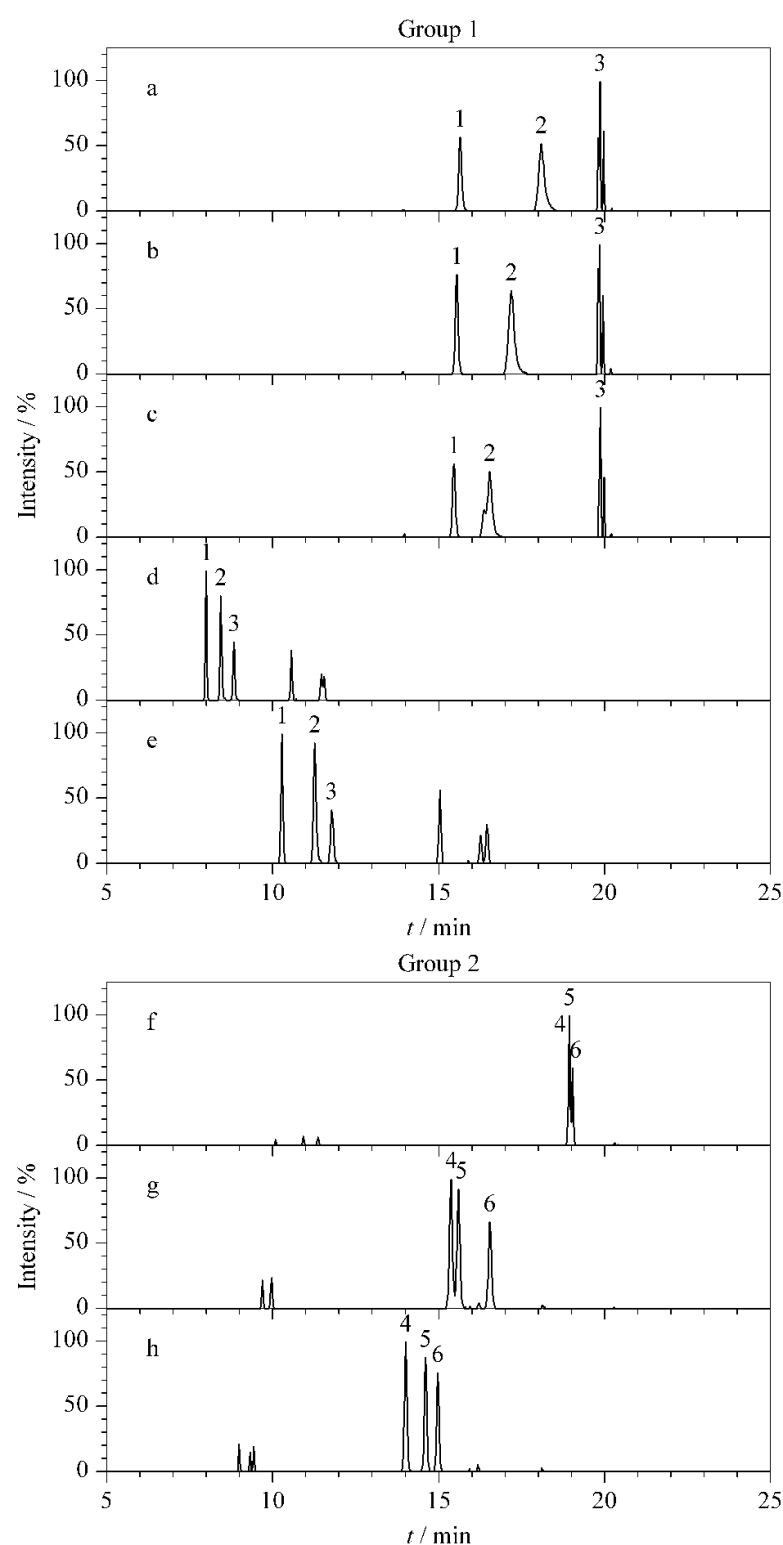
两组同分异构体的分离色谱图

在此基础上,继续考察流动相pH值对化合物色谱行为的影响,以同分异构体硫代豪莫西地那非、硫代艾地那非和丙氧苯基硫代西地那非为例:3种化合物的质荷比在正离子模式下均为505.2050,尝试添加不同体积分数的甲酸(包括添加0、0.01%、0.1%甲酸)改善同分异构体的分离。结果表明,随着甲酸体积分数的上升,3种化合物在更短的分析时间内获得了更好的分离效果,最终确定甲酸添加体积分数为0.1%,从而满足多组分高分辨质谱定量的需求,相关谱图见图1f~h。

研究表明,在上述流动相条件下,正、负离子扫描模式下各组分均获得有效分离,峰形良好,故确定为最终流动相。

### 2.2 前处理条件的选择

#### 2.2.1 提取溶剂的选择

首先从分配系数(oil-water partition coefficient,lg *P*)角度了解目标药物的溶解性,所有药物的lg *P*值为-8.5645~6.8819,其中98%的待测组分lg *P*<5,另外6种的lg *P*值为-5.1281~6.8819,均不属于强疏水性药物。同时,考虑到该值体现了液液萃取时的油水分配系数,而本研究需要从固体乳粉中直接将待测组分提取至混溶剂中,提取原理不尽相同,因此,本文选择对所有药物均具有良好溶解性的甲醇、乙腈以及乙腈高占比的水-乙腈 (2∶8,v/v)混合溶液作为提取溶剂进行实际考察,提取结果取决于是否能够有效脱脂、脱蛋白质,从而获得洁净即低离子干扰的提取物。分别以添加水平为10 μg/kg的牛源性和羊源性老年乳粉为基质(取试样2 g,置于50 mL具塞离心管中,分别加入20 μL 1 mg/L混合标准中间液和10 mL 40 ℃温水,涡旋1 min),比较了以下5种提取溶剂:乙腈、1%甲酸水溶液-乙腈(2∶8, v/v)、水-乙腈(2∶8, v/v)、0.1 mol/L Na_2_EDTA-Mcllvaine缓冲液-乙腈(2∶8, v/v)和甲醇。不同提取溶剂的效果见图2Ⅰ和2Ⅱ。

**图2:**
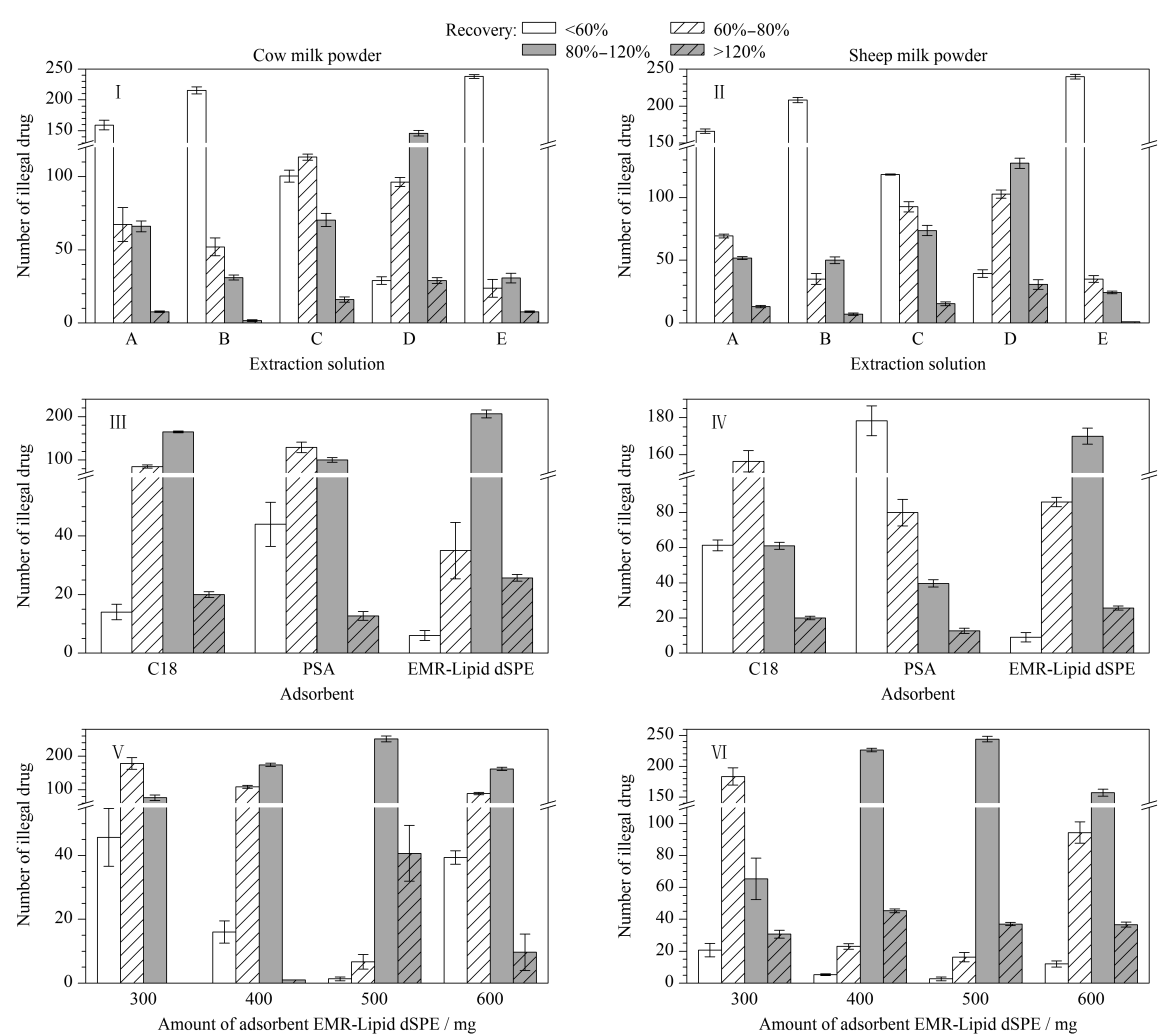
样品预处理条件优化(*n*=3)

当使用甲醇提取时,大部分目标化合物的回收率低于60%,可能由于乳粉中氨基酸、糖等大分子极性杂质易溶于甲醇,蛋白质沉淀效果较弱。当使用乙腈提取时,整体提取效率明显提高,可见乙腈的宽极性包容性为极性各异的多类化合物提供了较好的溶解度,而对蛋白质、脂肪、糖等干扰物的提取率低,因此选择乙腈作为提取溶剂的有机相。继续考察水相与有机相混合提取的效果及不同改性剂对提取效率的影响。将水、0.1%甲酸水溶液和0.1 mol/L Na_2_EDTA-Mcllvaine缓冲溶液分别与乙腈以2∶8的体积比混合作为提取溶液^[25]^。结果表明,与纯水配比的混合溶剂提取效率优于纯乙腈,但在水相中加入0.1%甲酸后部分化合物回收率明显降低,可能由于多数化合物在酸性条件下以离子形式存在,降低了其在有机溶剂中的溶解度。反之,与纯水相比,0.1 mol/L Na_2_EDTA-Mcllvaine缓冲液的加入,有效改善了沙星类等药物的回收率,说明此类药物可能与样品中的金属离子存在螯合作用,而Na_2_EDTA的加入有助于将药物释放。基于以上结果,确定0.1 mol/L Na_2_EDTA-Mcllvaine缓冲液-乙腈(2∶8, v/v)为提取溶剂。此时,共146种分析物在两种样品基质中的回收率在80%~120%范围内。可见,采用溶剂直接稀释法提取时,部分分析物的回收率仍不理想,有必要从基质净化角度进一步提高回收率。

#### 2.2.2 盐剂的优化

为了使目标化合物更完全地提取在乙腈层,本文按照1.3节下的处理流程操作,考察了4组盐剂的盐析效果,分别为0.5 g NaCl+2 g Na_2_SO_4_、0.5 g NaAc+2 g Na_2_SO_4_、0.5 g NaCl+2 g MgSO_4_和0.5 g NaAc+2 g MgSO_4_。结果表明,0.5 g NaCl+2 g Na_2_SO_4_的盐析效果最佳,188种分析物在两种样品基质中的回收率为80%~120%,全部300种分析物相应的回收率为40.3%~160.8%。在萃取过程中加入MgSO_4_会释放大量的热量,影响化合物的提取效率,且MgSO_4_容易吸水结块,影响样品均质效果。NaAc和MgSO_4_的加入降低了麻黄碱、沙丁胺醇等*β*-激动剂的回收率。因此,选用0.5 g NaCl+2 g Na_2_SO_4_作为两种样品基质的盐剂。

#### 2.2.3 吸附剂的优化

样品在提取过程中经过蛋白质沉淀后,仍可能存在一些脂肪杂质,由于脂质会引发质谱分析中的基质效应而使方法性能下降且仪器寿命缩短。因此,有必要进一步对提取液进行净化处理。考虑到目标组分的性质多样性,本研究选择QuEChERS吸附法,按照1.3节下的处理流程操作,分别考察了300 mg十八烷基键合硅胶(C18,北京迪马科技有限公司)、*N*-丙基乙二胺(PSA,北京迪马科技有限公司)和EMR-Lipid dSPE吸附剂对药物提取回收率的影响(如图2Ⅲ和2Ⅳ所示)。吸附效果为EMR-Lipid>C18>PSA。C18虽然具有去除脂肪和脂类等非极性干扰物的潜力,但同时也因为非选择性吸附造成了阿司匹林、氟比洛芬和巴比妥类目标物的损失。PSA通过阴离子交换吸附糖类和极性较高的酸性物质,对脂类物质吸附能力较差,且会影响喹诺酮类、阿托品、布洛芬等酸性药物的提取率。而增强型脂质去除材料EMR-Lipid能够选择性去除基质中的脂质而将待测组分保留在提取液中。以300 mg EMR-Lipid为吸附剂时,300种物质中68.7%的药物加标回收率保持在80%~120%,平均加标回收率为47.0%~159.1%,因此,最终选择EMR-Lipid作为吸附剂。继续对用量进行了研究,分别考察了EMR-Lipid使用量为300、400、500和600 mg时的净化效果。如图2Ⅴ所示,牛源性样品吸附剂用量与萃取效率差异的方差分析表明,使用400和500 mg EMR-Lipid时,萃取效率较其他两种用量显著提升(*P*<0.05);如图2Ⅵ所示,羊源性样品中吸附剂EMR-Lipid的使用量增加至500 mg时,提取效率显著提升(*P*<0.05)。推测原因为牛乳粉的脂肪含量略高于羊乳粉,因此,需要使用更多吸附剂去除脂类杂质。而继续增加吸附剂用量至600 mg,在两种样品基质中的回收率整体水平均呈现下降,最终选择EMR-Lipid吸附剂的使用量为500 mg。图3为空白样品经优化的QuEChERS方法处理前后的色谱图。

**图3:**
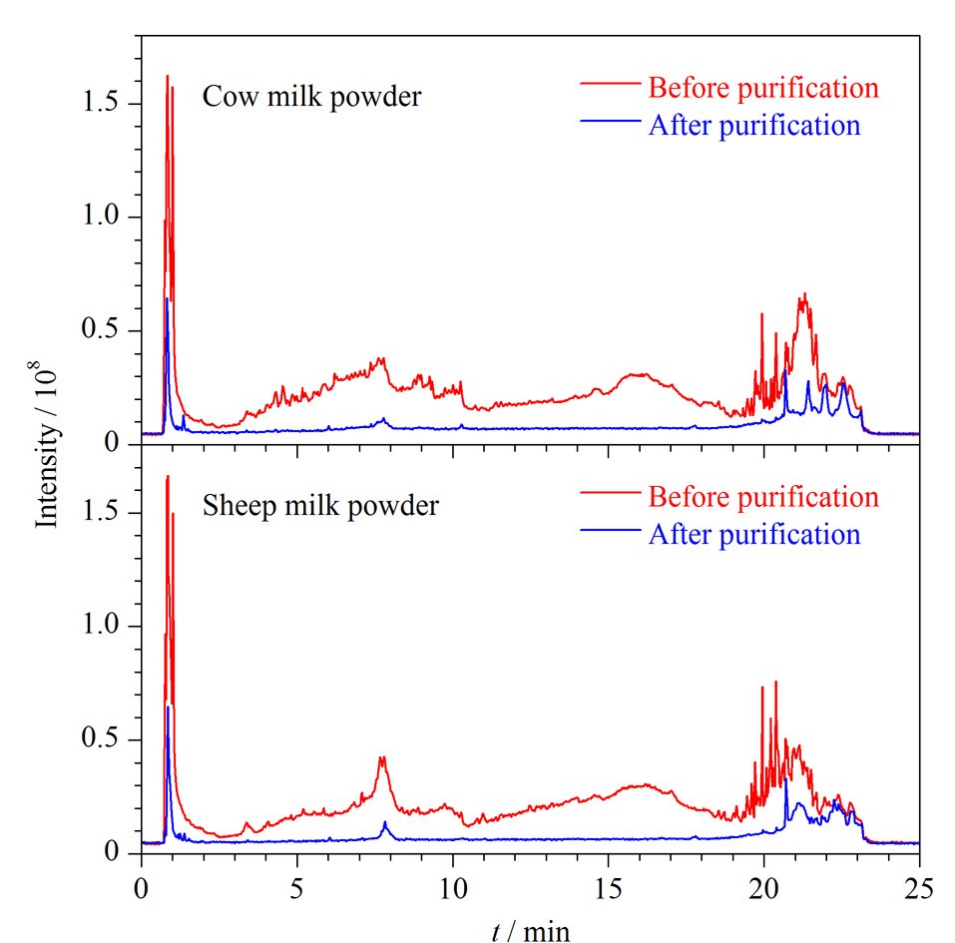
经优化的QuEChERS方法净化前后空白乳粉的色谱图

### 2.3 质谱条件的优化

#### 2.3.1 扫描方式的选择

基于Zeno SWATH^®^ DIA的UHPLC-Q-TOF/MS技术,对常见老年乳粉中300种非法添加化学药物进行鉴定及定量分析。首先,在两种电离模式下,比较了所有药物前体/产物离子的灵敏度,确定了更加敏感的加合方式及相应的正/负离子电离模式。大多数化合物的前体离子为质子化[M+H]^+^或去质子化[M-H]^-^分子离子,但达格列净、卡格列净选择[M+NH_4_]^+^峰进行分析能够获得更好的重复性结果,另外阿司匹林选择源内裂解峰作为母离子进行高灵敏度定量分析。

#### 2.3.2 质谱参数的优化

本研究利用Zeno TOF 7600系统的Zeno trap阱富集功能与TOF加速器离子脉冲相匹配,解决了传统Q-TOF系统低质量端离子利用率低的问题,从而提高了定量分析时的灵敏度和重复性。以苯乙双胍为例(见图4),优化后的定量峰面积增加了10倍以上,Zeno trap能够显著提升定量灵敏度。

**图4:**
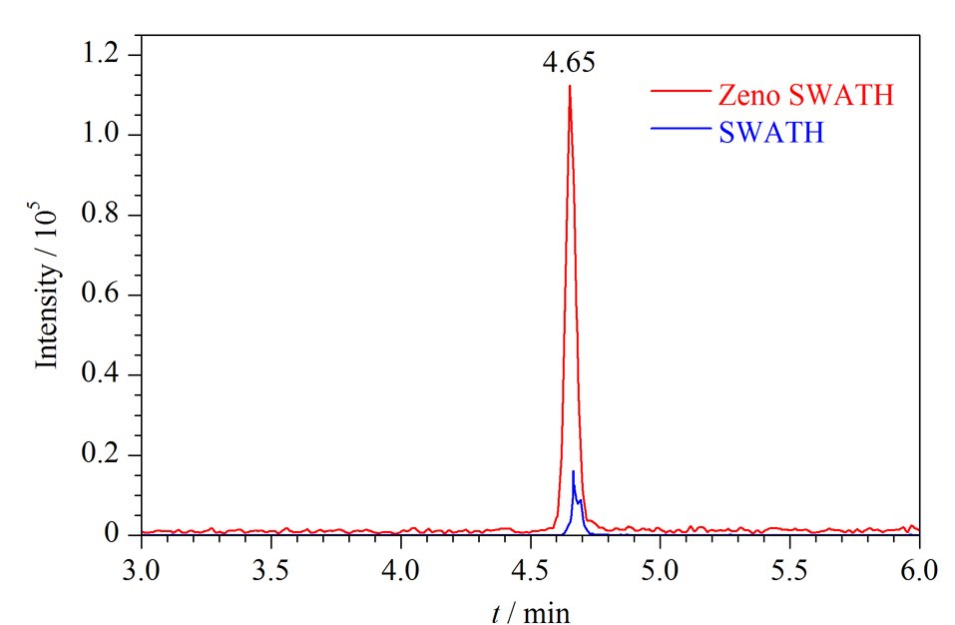
Zeno功能开启对苯乙双胍响应增加效果的色谱图

#### 2.3.3 基于谱库的定性筛查和确认

为了考察所建立的筛查方法的可靠性,本实验采用已建立的一级精确质量数据库和二级谱图库对添加了300种化学药物的乳粉样品进行自动检索。目标化合物的综合得分为91.9~99.8,所得结果均大于定性筛查的最低限(90分),筛查参数及结果分别见表1和附表1(www.chrom-China.com)。结果表明,本实验建立的筛查方法准确可靠,能够在无标准品的情况下完成所有目标药物的筛查与确证。

### 2.4 方法学评价

#### 2.4.1 选择性

在适当的保留时间窗口(RTW)内检索目标物,每个目标物的RTW为3倍空白加标样品(加标水平为10 μg/kg)保留时间标准差^[26,27]^。结果表明,空白样品在所有组分相应的RTW内均无干扰峰,检索到的目标分析物质量数的精确偏差范围≤5 ppm。

#### 2.4.2 基质效应(ME)

基质效应是基于电喷雾电离的质谱方法中最具挑战性的问题之一,通常通过比较标准物质在空白基质提取液和纯溶剂中的响应来评估。根据公式(ME=*k*_a_/*k*_b_, *k*_a_和*k*_b_分别为目标分析物在样品基质和溶剂中标准曲线的斜率)计算。300种目标物中*N*-苯丙烯基他达拉非、*N*-辛基去甲他达拉非、伐地那非乙酰基类似物等那非类物质以及培氟沙星、氟罗沙星、达氟沙星、恩诺沙星等沙星类药物的基质效应均大于1.5,可见基质干扰对质量精度和目标分析物响应的影响不能完全忽略。消除基质效应常用的方法有两种:基质匹配标准溶液法和同位素内标法。由于分析物种类繁多,性质各异,同位素内标不能完全对应。因此,本研究采用了基质匹配校准的方法来提高量化精度。

#### 2.4.3 线性范围、检出限和定量限

通过目标分析物的标准曲线(6点)考察方法的线性范围。在2种基质的相应线性范围内,所有组分均呈良好的线性关系,相关系数均大于0.99。以添加回收样品峰响应值为3倍噪声的添加水平为方法检出限(LOD),以10倍噪声的添加水平为方法定量限(LOQ)。羊乳粉和牛乳粉中所有分析组分的LOD分别为0.1~2.7 μg/kg和0.1~2.6 μg/kg。以两种样品基质中相对较高的LOQ确定方法的LOQ,LOQ为0.2~8.0 μg/kg,均优于我国现有标准中的灵敏度,可满足快速筛查及确证的需要。乳粉中300种化学药物的检出限、定量限、线性范围、基质匹配标准曲线和相关系数见附表2。

#### 2.4.4 回收率与精密度

通过加标回收率评价该方法的准确性。在低、中、高3个水平(1倍、5倍、20倍LOQ)下测定了加标样品中分析物的回收率,平行操作6次。77%分析物的平均回收率为80%~120%,如附表3所示,所有目标组分在乳粉中的回收率为73.1%~125.2%,相对标准偏差(RSD)≤14.8%。结果表明,该方法具有良好的准确性和精密度。

### 2.5 方法应用

应用本方法对60批样品中非法添加化学药物进行了快速筛查。经与数据库匹配分析,共检出2种非法添加物质:在1份声称调节血糖的老年乳粉中检出苯乙双胍,含量为1.4 mg/g。苯乙双胍曾被广泛用于临床Ⅱ型糖尿病的治疗(每日推荐剂量为25 mg)。但因该药会导致致死性乳酸酸中毒,且病死率极高,于2016年被我国禁止使用。而按照该乳粉推荐的每天食用量37.6 g计算,每日摄入量为41.4 mg,超过临床使用剂量的1.5倍;在1份添加人参等中药提取物的声称增强免疫力的样品中检出磷酸二酯酶选择性抑制剂西地那非,含量为3.9 mg/g。

相关谱图见图5。

**图5:**
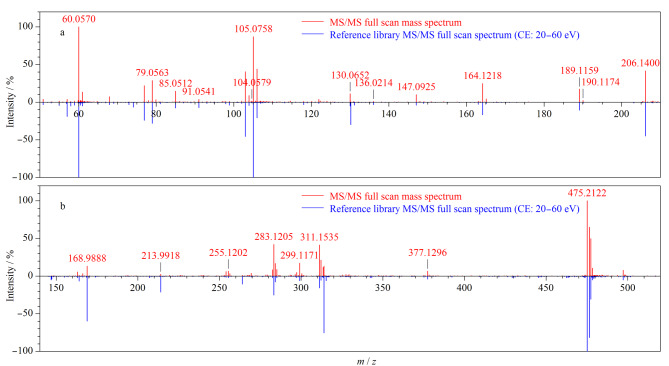
2个真实样品非法添加药物的鉴定谱图

此外,应用未知物的鉴定策略,本研究在1份声称调节血脂的老年乳粉中检出一种安非他明结构类似物。可疑物质在1.4节色谱条件下的保留时间为5.37 min,采用1.5节质谱条件进行一级质谱扫描,得到*m/z* 164.1434的准分子离子峰[M+H]^+^峰,经拟合后确定化合物的分子式为C_11_H_17_N (质量偏差0.0 ppm),进一步以*m/z* 164.1434为母离子进行子离子扫描,由二级碎片质谱图(图6a)可知,该化合物具有明显的与安非他明相同的特征子离子*m/z* 65.0386、91.0544、119.0855,说明其具有与安非他明相同的二级碎片结构。在此基础上,未知物的分子式相比安非他明增加一个-C_2_H_4_,因此推测该化合物结构可能是在安非他明的氨基上增加一个-C_2_H_4_,或者增加两个-CH_3_。经检索发现,两种疑似物结构分别为乙非他明和二甲基苯丙胺,互为同分异构体。通过进一步的二级碎片离子比对发现,在传统的CAD裂解模式下,两种化合物的二级碎片离子高度相似(图6b),通过二级碎片匹配基本无法区分。因此尝试采用EAD碎裂技术,将二者的母离子*m/z* 164.1434进行裂解,通过优化电子动能(10 eV)和电子流(9000 nA)可以得到更为丰富的二级碎片离子,如图6c所示,通过对比乙非他明和二甲基苯丙胺的二级碎片发现,二甲基苯丙胺在EAD模式下更容易断裂一个-CH_3_形成*m/z* 148.1127的碎片,而乙非他明在EAD模式下虽然可以断裂一个-CH_3_形成*m/z* 148.1126的碎片,但响应相对较低,其更容易断裂-CH_2_-CH_3_形成*m/z* 134.0971的碎片,获得在CAD模式下无法实现的两个特征碎片,因此可以对两种同分异构体进行快速区分。由此推测这份声称调节血脂的老年乳粉中检出的可疑未知物质为乙非他明,这种曾用于治疗肥胖症的药物因伴随厌食的严重副作用而撤出市场,目前作为第二类精神药品管理。经过与标准物质信息进行比对,保留时间和多级碎片离子均吻合,最终确认推测结果。

**图6:**
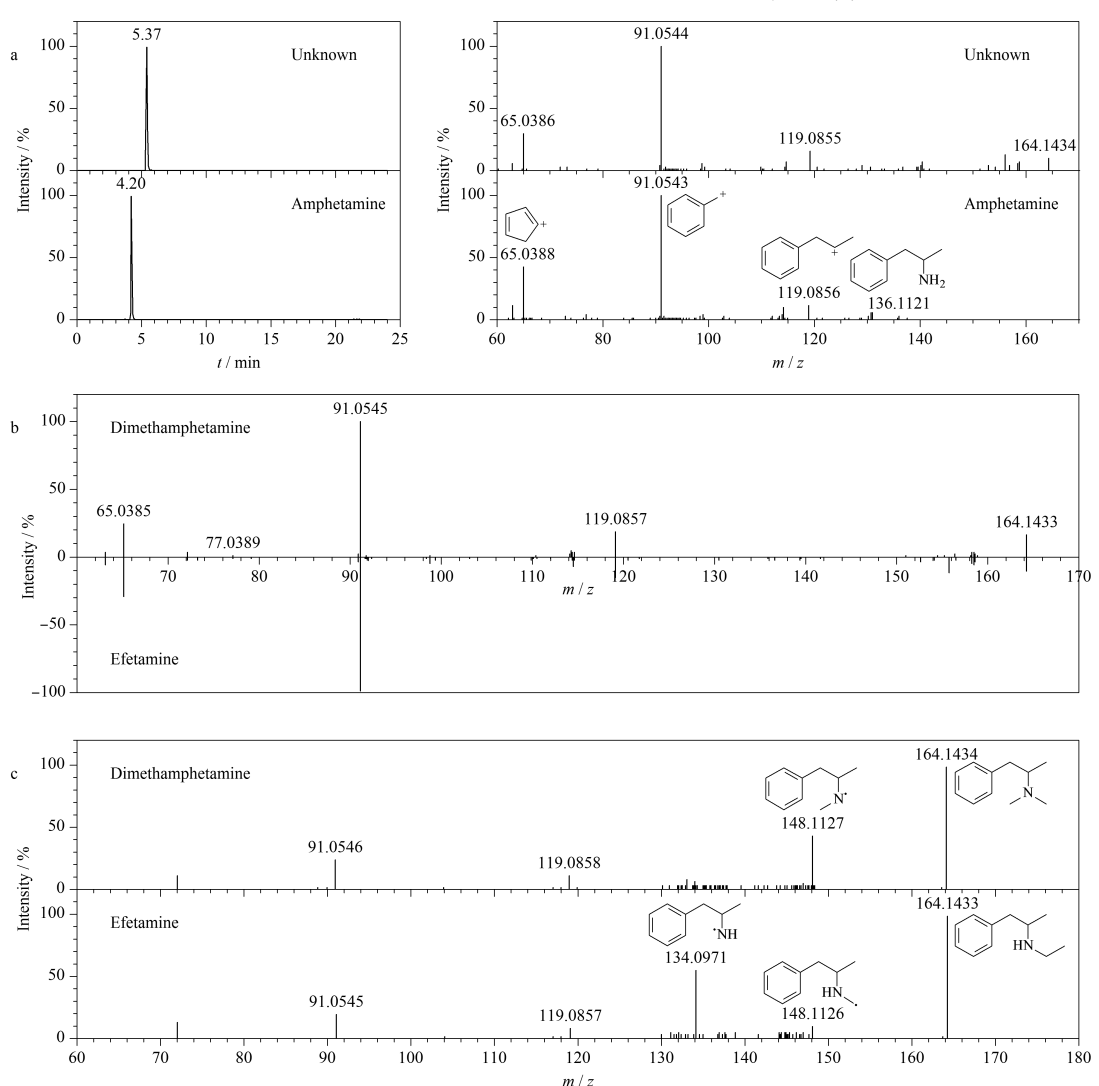
可疑样品中未知物的鉴定色谱图及质谱图

## 3 结论

本研究建立了一种基于QuEChERS提取结合SWATH模式的UHPLC-QTOF-MS分析方法,用于老年乳粉中300种非法添加化学药物的快速测定。该方法通过优化的QuEChERS提取净化方法,实现了从复杂样品基质中同时提取具有不同理化性质及疗效的多类别药物。优化后的方法在两种验证基质中的选择性、线性关系、灵敏度、基质效应、准确度和精密度结果令人满意,所有组分的分离检测在25 min内完成。在此基础上,采用EAD技术建立了一种未知物的非靶向筛查及鉴定策略。方法应用于不同地区市售真实样品的分析,在60份样品中检出苯乙双胍和西地那非2种非法添加药物,并鉴定出一种已撤出市场的减肥药品乙非他明,阳性样品检出率为5%。研究结果表明,新兴功能性乳粉可能对老年人存在潜在的健康风险,有必要进一步加大专项整治力度,扩大样品采集规模,全面开展涉老产品隐患排查。本研究建立的方法适用于多类别功能声称乳粉中300种化合物的高通量分析,同时为相关结构类似物的识别确证提供了一种有效的技术手段。
